# Identifying predictors of stroke in young adults: a machine learning analysis of sex-specific risk factors

**DOI:** 10.3389/fstro.2024.1488313

**Published:** 2024-11-18

**Authors:** Molly Jacobs, Noah Hammarlund, Elizabeth Evans, Charles Ellis

**Affiliations:** ^1^Department of Health Services Research, Management and Policy, College of Public Health and Health Professions, University of Florida, Gainesville, FL, United States; ^2^Department of Speech, Language and Hearing Science, College of Public Health and Health Professions, University of Florida, Gainesville, FL, United States

**Keywords:** young stroke, machine learning (ML), sex, risk, behavior

## Abstract

**Introduction:**

Stroke among Americans under age 49 is increasing. While the risk factors for stroke among older adults are well-established, evidence on stroke causes in young adults remains limited. This study used machine learning techniques to explore the predictors of stroke in young men and women.

**Methods:**

The least absolute shrinkage and selection operator algorithm (LASSO) was applied to data from Wave V of the National Longitudinal Survey of Adolescent to Adult Health (*N* = 12,300)—nationally representative, longitudinal panel containing demographic, lifestyle, and clinical information for individuals aged 33–43—to identify the key factors associated with stroke in men and women. The resulting LASSO model was tested and validated on an independent sample and model performance was assessed using the area under the receiver operating characteristic curve (AUC) and calibration. For robustness, synthetic minority over sampling technique (SMOTE) was applied to address data imbalance and analyses were repeated on the balanced sample.

**Results:**

Approximately 1.1% (*N* = 59) and 1.3% (*N* = 90) of the 5,318 and 6,970 men and women in the sample reported having a stroke. LASSO was used to predict stroke using demographic, lifestyle, and clinical predictors on both balanced and imbalanced data sets. LASSO performed slightly better on the balanced data set for women compared to the unbalanced set (Female AUC: 0.835 vs. 0.842), but performance for men was nearly identical (Male AUC: 0.820 vs. 0.822). Predictor identification was similar across both sets. For females, marijuana use, receipt of health services, education, self-rated health status, kidney disease, migraines, diabetes, depression, and PTSD were predictors. Among males, income, kidney disease, heart disease, diabetes, PTSD, and anxiety were risk factors.

**Conclusions:**

This study showed similar clinical risk factors among men and women. However, variations in the behavioral and lifestyle determinants between sexes highlight the need for tailored interventions and public health strategies to address sex-specific stroke risk factors among young adults.

## Introduction

An estimated 10%−15% of all first-ever strokes occur in people aged 18–50 years (Kissela et al., 2012; Singhal et al., 2013). With a yearly stroke incidence of 15 million people worldwide, at least 1.5 million young adults are affected every year. While the incidence of stroke among the elderly is declining, stroke in younger adults is increasingly common (Wilson and Biller, 2004; Aigner et al., 2017). Additionally, young adults are less likely to die from stroke than older adults and their risk is significantly higher than that of the age-adjusted general population (Bukhari et al., 2023). One-third of young adult stroke survivors are left with moderate to severe functional impairment (Varona et al., 2004) and 40% have long-term cognitive impairment (Jia, 2015). Stroke at a young age not only results in impairment in basic daily activities but also impacts participation in normal activities, such as returning to work, family, and social activities (Treger et al., 2007; Pollock et al., 2014).

Young stroke survivors are more likely to experience marital problems such as separation or divorce (Teasell et al., 2000; Banks and Pearson, 2004), unmet financial need due to the number of productive life years lost (Sultan and Elkind, 2013; Béjot et al., 2016), and long-term sequelae including permanent cognitive deficits, epilepsy, and chronic debilitating fatigue with poor functional outcomes (Schaapsmeerders et al., 2013; Maaijwee et al., 2015; Arntz et al., 2013; Amoah et al., 2024). The social and economic burdens of stroke in young adults are substantial due to loss of prime productive years, longer time spent with disability, and increased mortality (Ekker et al., 2019). More specifically, strokes in younger adults carry the potential for a greater lifetime burden of disability and may have more catastrophic consequences for people of working age (Vestling et al., 2003). Finally, stroke in young adults is more challenging because the variability in clinical presentation and differences relative to older adults (Bukhari et al., 2023).

In addition to age-related variation in stroke and stroke-related outcomes, research also indicates sex differences in the both the incidence and risk factors associated with stroke (Lasek-Bal et al., 2018). Although individual studies vary in their findings, a recent meta-analysis revealed that young adult women experience strokes at a 44% higher rate than men (Leppert et al., 2022). Additionally, Reeves et al. (2008) found that traditional stroke risk factors like hypertension and diabetes had different impacts on stroke risk in men and women, necessitating sex-specific preventive strategies. Moreover, a study by Bushnell et al. (2014) emphasized the importance of considering sex differences in stroke risk to improve the accuracy of predictive models and the effectiveness of interventions. These findings underscored the need for separate analysis to develop more precise, sex-specific public health policies and individualized treatment plans that can better address the unique risk factors and improve outcomes for both young men and women.

One potential area that has shown promise in better understanding stroke in young adults is related to the use of machine learning to explore variations in stroke outcomes (Chandrabhatla et al., 2023). Machine learning (ML) is a data analytics methodology that is increasingly being used to explore the relationship between and make predictions of outcomes derived from multiple data sources (Ni et al., 2018). ML uses algorithms that iteratively learn from multiple inputs of training data to determine complex relationships within the data to improve prediction on future data sources (Ni et al., 2018). ML approaches have been utilized in stroke diagnosis (Mainali et al., 2021; Dev et al., 2022), stroke risk factor identification (Hassan et al., 2024), stroke imaging (Sheth et al., 2023; Soun et al., 2021), and stroke outcome prediction (Mainali et al., 2021). Given the substantial contributions of ML to the study of stroke overall, ML approaches appear ideal to study stroke in young adults to improve diagnosis, treatment and patient-related outcomes (Daidone et al., 2024). Therefore, this study was designed to examine sex-specific risk factors among young adults with stroke. The team used a nationally representative sample of young adults aged 33–43 (Lee et al., 2022). Predictive modeling was utilized to identify the key sex-specific stroke risk factors and how these risk factors varied between young men and women with the ultimate goal of characterizing the multifactorial nature of stroke incidence in younger adults.

## Materials and methods

### Data

Data for this study came from the National Longitudinal Survey of Adolescent Health (ADD Health)—a nationally representative, longitudinal survey of individuals who were in Grades 7–12 during the 1994–1995 school year in the United States. ADD Health includes longitudinal data on respondents' social, economic, psychological and physical well-being with contextual data on the family, neighborhood, community, schools, friendships, peer groups, and romantic relationships, providing unique opportunities to study how health, social environments, and behaviors are linked over time. The initial Wave I sample (*N* = 20,745) represented the national cohort of adolescents in grades 7 to 12 in the US in 1995. This cohort was followed into young adulthood with five in-home interviews in 1995 (Wave I), 1996 (Wave II, *N* = 17,738), 2001–2002 (Wave III, *N* = 15,197), 2008–09 (Wave IV, *N* = 15,701), and 2016–18 (Wave V, *N* = 12,300) when respondents were 12–17, 13–18, 18–26, 24–32, and 33–43 years old, respectively. Each wave consisted of core household, demographic and health information along with additional wave-specific topics.

This study used data collected in Wave V (*n* = 12,300) when all respondents were age 18 and above as well as information reported in the Wave I parental survey. Wave V collected social, environmental, behavioral, and biological data with which to track the emergence of chronic disease as the cohort advanced through their 30s and early 40s. The data collection employed a mixed mode survey design consisting of web, in-person, telephone, and mail-based questionnaires and interviews (Harris et al., 2019). Additional information on the sampling design, survey modes, instrumentation, and validation can be found here https://addhealth.cpc.unc.edu/wp-content/uploads/docs/user_guides/Add-Health-Wave-V-Sampling-and-Mixed-Mode-Survey-Design_doi.pdf. They Wave V survey was the first to include questions related to stroke and family history of stroke. Survey items included in this study are described below. Using these data, we sought to identify specific predictive factors associated with stroke among young men and women. To explore the influence of data balancing methods on the performance of the LASSO, analyses were performed on both the balanced and imbalanced data set for both sexes.

### Outcome variables

In Wave V, respondents indicated whether a doctor, nurse, or health care provider diagnosed them with a stroke. Responses were coded as either zero which represented no prior stroke diagnosis or one which represented a prior stroke diagnosis. Respondents who asked for additional clarity were told to respond affirmatively if they had been diagnosed with a stroke, ministroke, or received surgery for clogged neck arteries (including endarterectomy, bypass, angioplasty, or stent).

### Candidate variables

Candidate variables included important confounding variables in stroke risk, but excluded those that were themselves potential outcomes of stroke since their inclusion would bias the focal association (Hernan, 2002; Pearl, 2009; Elwert, 2013). Therefore, to capture factors associated with stroke (Cramer and Kapusta, 2017), sets of theoretically relevant demographic, lifestyle, and clinical characteristics were chosen from those available in the ADD Health database.

### Demographic factors

Demographic factors included age, sex at birth (male, female), race, ethnicity, highest educational attainment (less than a college degree, college degree or above), employment status (currently working 10+ hours per week, not working 10+ hours per week), marital status (married, not married), school enrollment (currently enrolled in an educational degree program at least part-time, not enrolled), and income level (< $75,000, ≥$75,000). Race was self-reported as White, Black, Asian or Pacific Islander, American Indian or Native American, or other. Due to sample size limitations, Asian or Pacific Islander, American Indian or Native American, and other were collapsed into a single category. Ethnicity was self-reported as either Hispanic or non-Hispanic. To account for early life and familial characteristics, indicators for parental education (high school diploma or above), parental marital status (married), and parental income (≥$75,000) were created. Additional indicators were created for residing in the South, Midwest, or West and living within or near a neighborhood area historical classified as “definitely declining” or “hazardous” by the Homeowners Loan Corporation (HOLC)—also known as a historically “red” neighborhood. Finally, indicators for prior stroke diagnosis among biological aunts/uncles, grandparents, parents, and siblings were created.

### Lifestyle factors

Indicators were created from survey items capturing the frequency of engaging in health-impacting behaviors. Binary behavioral indicators were created from ordinal survey variables based on the univariate distributions. These indicators included consuming alcohol more than once monthly, smoking at least one cigarette monthly, used marijuana at least once in the past month, watching more than 20 h of television weekly, and exercising at least once weekly. Responses to individual survey items concerning use of illicit drugs including cocaine, crystal meth, heroin, or other types of illegal drugs, such as LSD, PCP, ecstasy, or mushrooms or inhalants in the last month were combined into a single variable. Finally, an additional indicator for the use of prescription sedatives, tranquilizers, stimulants, or pain killers that were not prescribed, taken in larger amounts than prescribed, more often than prescribed, for longer periods than prescribed, or taken for the feeling or experience they caused in the past 30 days was created.

### Clinical factors

Health-related characteristics included self-reported health status, health services utilization, and diagnoses. Indicators for self-reported good/very good/excellent health, being obese (body mass index ≥30), having health insurance, and self-reported diagnosis of diabetes, heart disease, migraine headaches, kidney disease/kidney failure, depression, anxiety, hyperlipidemia, or high blood pressure were included. Additionally, receipt of health services was captured using indicators for receipt of mental health counseling within the last 12 months, taking at least one prescription medication regularly, having a dental exam within the last 12 months, having a regular doctor or health center, and having not received needed health services in the last year. For female respondents, indicators for taking oral contraception, and having previously had at least one live birth were created.

### Data analysis approach

As previously indicated, continuous and ordinal variables were transformed into categorical outcomes using established or pragmatic thresholds to enhance interpretability and simplify interpretation of findings (Bennette and Vickers, 2012; Barrio et al., 2017). Many variables have been reported to influence stroke occurrence including age, sex, race/ethnicity, family history, genetic factors, hypertension, diabetes, heart disease, high cholesterol, smoking, obesity, physical inactivity, diet, alcohol and substance use, previous stroke or transient ischemic attack (TIA), sleep apnea, hormonal factors (e.g., oral contraceptives, hormone replacement therapy), chronic stress, socioeconomic status (Yahya et al., 2020). To identify factors associated with young stroke, predictive modeling techniques were employed to uncover the most important predictors from a complex dataset. This approach is vital as it identifies key risk factors while allowing for more accurate model generalization. We examined a dataset encompassing 51 and 53 variables for men and women, respectively, the unequal number resulting from several female specific characteristics related to pregnancy and childbirth. This dataset included a spectrum of demographic, lifestyle, and clinical information. However, the relationship between various social, behavioral, and health-related outcomes often requires advanced approaches to identify the most important predictors without overfitting which cannot be easily rectified using standard techniques (Irvin et al., 2020; Richmond et al., 2020; Kino et al., 2021).

Regularization, a technique designed to generalize models in the context with many potentially important predictors, was completed by adding a penalty to model parameters. This approach helps the model generalize to the data rather than overfitting to the training set. Least Absolute Shrinkage Selector Operator (LASSO), a type of regularization, was utilized to minimize model overfitting by applying a penalty term (λ) to the log-likelihood function and setting the coefficients of unimportant predictors to zero (Tibshirani, 1996). LASSO simultaneously performs variable selection by identifying the most important predictors while managing model complexity. This is particularly valuable in datasets with numerous predictors, thereby enhancing our understanding of stroke risk among young adults. The approach has been used in a variety of settings with complex sets of underlying predictors (Ortega Hinojosa et al., 2014; Simeonov and Himmelstein, 2015). LASSO was executed using the *glmnet* package (Tay et al., 2023; Friedman et al., 2010) in R software (R Core Team, 2021) (version 4.4.0), incorporating a ten-fold cross-validation strategy to ascertain the optimal regularization parameter (λ). A random training set (70%) was selected to train the modes and a random hold-out test set (30%) to assess its performance. To ensure model results were not influenced by multicollinearity between factors, variance inflation factors (VIF) were inspected. All VIFs were below five suggesting a low correlation with other predictors.

On the training set, 10 × 10-fold cross validation was used to select the optimal lambda value within one standard error of the minimal cross-validation error (i.e., lambda.1se criterion) (Tibshirani, 1996). Through this procedure, we identified key indicators that manifested non-zero coefficients in the LASSO model and were identified as predictors of stroke occurrence among men and women.

To ensure that poor data quality did not degrade the final prediction, data discretization, redundant values reduction, and class balancing was performed to make it more appropriate for mining and analysis (Fan et al., 2021). Class balancing employed the synthetic minority oversampling technique (SMOTE) (Maldonado et al., 2019) to address the imbalanced distribution of participants among the stroke and non-stroke classes. SMOTE was executed using the *performanceEstimation* (Torgo, 2014) package in R and generated synthetic samples by oversampling the minority class to balance the class distribution. However, since balancing a dataset can itself introduce bias (Krawczyk, 2016), analyses were conducted with both the unbalanced (original) and balanced data.

To interpret the results from the LASSO regression model, the magnitude of the coefficients was used to determine the strength of the association with larger magnitudes indicating a stronger association or more predictive value while the sign of the coefficient indicated the direction of the association (Wiemken and Kelley, 2020). To evaluate the performance of the model, the model prediction was tested using the testing data set then the model AUC, accuracy, precision, and recall were calculated (Friedman et al., 2010).

## Results

Among 12,300 respondents in the sample, 6,970 (56.67%) were female and 53,18 (43.53%) were male with mean ages of 37.45 (SD = 1.88) and 37.71 (SD = 1.89), respectively. About one percent of the sample (*N* = 149, 90 female, 49 male) reported having a stroke. Comparison of demographic, lifestyle, and clinical characteristics of the female and male samples is shown in Table 1. Comparisons of the stroke cohort and the cohort without stroke and within each sex is shown in Tables 2, 3. There were few differences between the balanced and unbalanced cohorts. For ease of interpretability, cohort comparison results were based on the unbalanced sample. The percent of the stroke cohort with hypertension (male 32.2%; female 27.78%), diabetes (male 25.42%; female 21.11%), kidney disease (male 18.64%; female 14.44%), chronic migraines (male 23.73%; female 75.56%), hyperlipidemia (male 38.98%; female 24.44%), and obesity (male 55.93%; female 55.56%) was significantly higher compared to the non-stroke cohort for both the male and female samples. Similarly, the portion of the male and female stroke cohorts reporting marijuana (male 32.20%; female 31.11%), illegal drug (male 3.39%; female 8.89%), and cigarette (male 40.68%; female 35.56%) usage was also higher than their non-stroke counterparts.

**Table 1:** Demographic, lifestyle, and clinical characteristics by sex.

	**Female (*****N*** = **6,970, 56.67%)**		**Males (*****N*** = **5,318, 43.53%)**		**Sex difference**	
	**Mean**	**SD**	**Mean**	**SD**	***F*** **-stat**	**Prob**
Age (33–43)	37.45	1.88	37.71	1.89	1.01	0.66
Live births (0–9)	1.69	1.33				
	***N***	**Percent**	***N***	**Percent**	χ^2^	***p*** **-Value**
Stroke	90	1.29	59	1.11	0.83	0.36
Good self-reported health	3,755	53.87	2,745	51.62	6.16	0.01
Hypertension	1,183	16.97	1,316	24.75	112.51	< 0.0001
Diabetes	421	6.04	205	3.85	29.80	< 0.0001
Kidney disease/failure	60	0.86	51	0.96	0.32	0.57
Heart disease	88	1.26	76	1.43	0.64	0.43
Chronic migraines	2,372	34.03	857	16.12	499.83	< 0.0001
Hyperlipidemia	1,020	14.63	1,117	21	85.20	< 0.0001
Obese	2,991	42.91	2,264	42.57	0.14	0.71
Depression	2,544	36.5	1,134	21.32	331.23	< 0.0001
Anxiety	2,143	30.75	902	16.96	307.51	< 0.0001
PTSD	525	7.53	293	5.51	19.86	< 0.0001
Dental appointment in past 12 months	4,745	68.08	3,124	58.74	114.11	< 0.0001
Counseling within last 12 months	1,145	16.43	633	11.9	49.90	< 0.0001
Health insurance	6,496	93.2	4,798	90.22	35.97	< 0.0001
Has regular health facility	4,208	60.37	2,538	47.72	194.90	< 0.0001
Did not received necessary care last 12 months	1,592	22.84	1,091	20.52	9.56	0.00
Takes ≥1 prescription medication	1,786	25.62	863	16.23	157.49	< 0.0001
Takes oral contraception	3,854	55.29				
Mother had a stroke	10	0.14	3	0.06	2.16	0.14
Father had a stroke	10	0.14	3	0.06	2.16	0.14
Sibling(s) had a stroke	9	0.13	6	0.11	0.07	0.80
Aunt(s)/uncle(s) had a stroke	114	1.64	89	1.67	0.03	0.87
Grandparent(s) had a stroke	206	2.96	163	3.07	0.12	0.72
Parents Married	6,963	99.9	5,315	99.94	0.72	0.40
Parents education ≥ high school	5,915	84.86	4,569	85.92	2.67	0.10
Parents earned >$75,000	629	9.02	533	10.02	3.51	0.06
Hispanic	1,040	14.92	789	14.84	0.02	0.90
Black	1,597	22.91	952	17.9	46.07	< 0.0001
Other race	551	7.91	488	9.18	6.30	0.01
Education level college degree or above	2,909	41.74	1,776	33.4	88.94	< 0.0001
Married	4,019	57.66	3,109	58.46	0.79	0.37
Household income >$75,000	2,554	36.64	2,836	53.33	341.06	< 0.0001
Currently employed	5,614	80.55	4,689	88.17	129.55	< 0.0001
Currently enrolled in school at least part-time	635	9.11	338	6.36	31.39	< 0.0001
Exercise ≥1 time weekly	4,746	68.09	3,082	57.95	134.08	< 0.0001
Used marijuana ≥1 time last month	1,047	15.02	1,197	22.53	113.63	< 0.0001
Used illegal drugs ≥1 time last month	179	2.57	247	4.64	38.77	< 0.0001
Improperly used prescription medication	796	11.42	557	10.46	2.80	0.09
Smokes Regularly	1,444	20.72	1,395	26.23	51.63	< 0.0001
Consumes alcohol ≥1 time weekly	3,274	46.97	3,202	60.21	212.06	< 0.0001
Watches ≥20 h of TV weekly	1,034	14.84	968	18.2	25.08	< 0.0001
HOLC grade declining or hazardous	2,298	32.97	1,753	32.96	0.00	0.99
South	2,923	41.94	2,140	40.24	3.58	0.06
Midwest	1,599	22.94	1,236	23.24	0.15	0.70
West	1,649	23.66	1,237	23.26	0.27	0.61

**Table 2:** Female cohort demographic, lifestyle, and clinical characteristics by stroke status.

	**No stroke (*****N*** = **6,880)**		**Stroke (*****N*** = **90)**		**Difference**	
	**Mean**	**SD**	**Mean**	**SD**	***F*** **-stat**	**Prob**
Age	37.45	1.88	37.69	1.88	1.00	1.00
Live births	1.69	1.33	1.93	1.45	1.19	0.21
	***N***	**Percent**	***N***	**Percent**	χ^2^	***p*** **-Value**
Good self-reported health	3,737	54.32	18	20	42.10	< 0.0001
Hypertension	1,158	16.83	25	27.78	7.55	0.01
Diabetes	402	5.84	19	21.11	36.49	< 0.0001
Kidney disease/failure	47	0.68	13	14.44	197.13	< 0.0001
Heart disease	77	1.12	11	12.22	87.85	< 0.0001
Chronic migraines	2,304	33.49	68	75.56	70.03	< 0.0001
Hyperlipidemia	998	14.51	22	24.44	7.02	0.01
Obese	2,941	42.75	50	55.56	5.95	0.01
Depression	2,483	36.09	61	67.78	38.49	< 0.0001
Anxiety	2,091	30.39	52	57.78	31.29	< 0.0001
PTSD	503	7.31	22	24.44	37.44	< 0.0001
Dental appointment in past 12 months	4,699	68.3	46	51.11	12.08	0.00
Counseling within last 12 months	1,117	16.24	28	31.11	14.32	0.00
Health insurance	6,417	93.27	79	87.78	4.23	0.04
Has regular health facility	4,157	60.42	51	56.67	0.52	0.47
Did not received necessary care last 12 months	1,553	22.57	39	43.33	21.73	< 0.0001
Takes ≥1 prescription medication	1,750	25.44	36	40	9.89	0.00
Takes oral contraception	3,803	55.28	51	56.67	0.07	0.79
Mother had a stroke	9	0.13	1	1.11	5.96	0.01
Father had a stroke	9	0.13	1	1.11	5.96	0.01
Sibling(s) had a stroke	8	0.12	1	1.11	6.82	0.01
Aunt(s)/uncle(s) had a stroke	112	1.63	2	2.22	0.20	0.66
Grandparent(s) had a stroke	199	2.89	7	7.78	7.39	0.01
Parents earned >$75,000	623	9.06	6	6.67	0.62	0.43
Parents education ≥ high school	5,837	84.84	78	86.67	0.23	0.63
Parents Married	6,873	99.9	0	0	0.09	0.76
Hispanic	1,032	15	8	8.89	2.61	0.11
Black	1,574	22.88	23	25.56	0.36	0.55
Other race	546	7.94	5	5.56	0.69	0.41
Education level college degree or above	2,892	42.03	17	18.89	19.57	< 0.0001
Married	3,973	57.75	46	51.11	1.60	0.21
Household income >$75,000	2,537	36.88	17	18.89	12.38	0.00
Currently employed	5,556	80.76	58	64.44	15.08	0.00
Currently enrolled in school at least part-time	625	9.08	10	11.11	0.44	0.51
Exercise ≥1 time weekly	4,683	68.07	63	70	0.15	0.70
Used marijuana ≥1 time last month	1,019	14.82	28	31.11	18.46	< 0.0001
Used illegal drugs ≥1 time last month	171	2.49	8	8.89	14.56	0.00
Improperly used prescription medication	778	11.31	17	18.89	5.05	0.02
Smokes regularly	1,412	20.52	32	35.56	12.22	0.00
Consumes alcohol ≥1 time weekly	3,238	47.06	36	40	1.78	0.18
Watches ≥20 h of TV weekly	1,017	14.78	17	18.89	1.19	0.28
HOLC grade declining or hazardous	2,267	32.95	31	34.44	0.09	0.76
South	2,889	41.99	34	37.78	0.65	0.42
Midwest	1,568	22.79	31	34.44	6.82	0.01
West	1,634	23.75	15	16.67	2.47	0.12

**Table 3:** Male cohort demographic, lifestyle, and clinical characteristics by stroke status.

	**No stroke (*****N*** = **5,259)**		**Stroke (*****N*** = **59)**		**Difference**	
	**Mean**	**SD**	**Mean**	**SD**	***F*** **-stat**	**Prob**
Age	37.71	1.89	37.95	2.03	1.15	0.4003
	***N***	**Percent**	***N***	**Percent**	χ^2^	***p*** **-Value**
Good self-reported health	2,730	51.91	15	25.42	16.39	< 0.0001
Hypertension	1,297	24.66	19	32.2	1.78	0.18
Diabetes	190	3.61	15	25.42	74.89	< 0.0001
Kidney disease/failure	40	0.76	11	18.64	196.46	< 0.0001
Heart disease	64	1.22	12	20.34	151.45	< 0.0001
Chronic migraines	843	16.03	14	23.73	2.56	0.11
Hyperlipidemia	1,094	20.8	23	38.98	11.62	0.00
Obese	2,231	42.42	33	55.93	4.36	0.04
Depression	1,105	21.01	29	49.15	27.54	< 0.0001
Anxiety	875	16.64	27	45.76	35.14	< 0.0001
PTSD	280	5.32	13	22.03	31.29	< 0.0001
Dental appointment in past 12 months	3,098	58.91	26	44.07	5.30	0.02
Counseling within last 12 months	619	11.77	14	23.73	7.96	0.00
Health insurance	4,741	90.15	57	96.61	2.76	0.10
Has regular health facility	2,510	47.73	28	47.46	0.00	0.97
Did not received necessary care last 12 months	1,074	20.42	17	28.81	2.52	0.11
Takes ≥1 prescription medication	844	16.05	19	32.2	11.20	0.00
Takes oral contraception	0	0	0	0	0.03	0.85
Mother had a stroke	3	0.06	0	0	0.03	0.85
Father had a stroke	3	0.06	0	0	0.03	0.85
Sibling(s) had a stroke	6	0.11	0	0	0.07	0.80
Aunt(s)/uncle(s) had a stroke	86	1.64	3	5.08	4.22	0.04
Grandparent(s) had a stroke	160	3.04	3	5.08	0.82	0.37
Parents earned >$75,000	529	10.06	4	6.78	0.70	0.40
Parents education ≥ high school	4,516	85.87	53	89.83	0.76	0.38
Parents Married	5,256	99.94	59	100	0.03	0.85
Hispanic	783	14.89	6	10.17	1.03	0.31
Black	934	17.76	18	30.51	6.45	0.01
Other race	483	9.18	5	8.47	0.04	0.85
Education level college degree or above	1,763	33.52	13	22.03	3.46	0.06
Married	3,083	58.62	26	44.07	5.09	0.02
Income >$75,000	2,821	53.64	15	25.42	18.67	< 0.0001
Currently employed	4,653	88.48	36	61.02	42.19	< 0.0001
Currently enrolled in school at least part-time	334	6.35	4	6.78	0.02	0.89
Exercise ≥1 time weekly	3,047	57.94	35	59.32	0.05	0.83
Used marijuana ≥1 time last month	1,178	22.44	19	32.2	3.18	0.00
Used illegal drugs ≥1 time last month	245	4.66	2	3.39	0.21	0.65
Improperly used prescription medication	546	10.38	11	18.64	0.25	0.04
Smokes regularly	1,371	26.07	24	40.68	6.43	0.01
Consumes alcohol ≥1 time weekly	3,179	60.45	23	38.98	11.22	0.00
Watches ≥20 h of TV weekly	952	18.1	16	27.12	3.19	0.07
HOLC grade declining or hazardous	1,731	32.92	22	37.29	0.50	0.48
South	2,107	40.06	33	55.93	6.11	0.01
Midwest	1,223	23.26	13	22.03	0.05	0.83
West	1,230	23.39	7	11.86	4.34	0.04

Table 4 provides results from predictive models of stroke risk including model performance, accuracy, feature selection, and feature coefficients. Of the 51 and 53 variables entered in the LASSO for the female and male cohorts, respectively, results showed that 10 (balanced 6) and 8 (balanced 6) were associated with stroke in the female and male unbalanced data sets. The most predictive clinical features for males were kidney disease, heart disease, and diabetes, and the most important clinical variables among females were kidney disease, chronic migraines, diabetes, and depression. The AUC values for the balanced and imbalanced data models were similar, indicating consistent predictor identification and model performance. Mental health variables were also identified as predictors of stroke—PTSD (Sumner et al., 2023) and anxiety (Ryder and Cohen, 2021) for males and PTSD (Ebrahimi et al., 2021) and depression for females (Dong et al., 2012).

**Table 4:** Model performance and feature selection.

**Females**				**Males**			
**Female unbalanced**		**Female balanced**		**Male unbalanced**		**Male balanced**	
BD	0.11	BD	0.47	BD	0.11	BD	0.44
ME	0.01	ME	0.08	ME	0.01	ME	0.08
AUC	0.84	AUC	0.83	AUC	0.82	AUC	0.82
MSE	0.02	MSE	0.13	MSE	0.02	MSE	0.12
MAE	0.05	MAE	0.26	MAE	0.04	MAE	0.25
Intercept	−5.06	Intercept	−2.95	Intercept	−4.77	Intercept	−2.79
Kidney disease	1.96	Kidney disease	1.05	Kidney disease	2.24	Kidney disease	1.92
Diabetes	0.80	Chronic migraines	0.69	Heart disease	2.05	Diabetes	1.65
Chronic migraines	0.76	Diabetes	0.63	Diabetes	0.41	PTSD	0.44
Depression	0.24	Depression	0.15	Anxiety	0.38	Heart disease	0.38
Used marijuana	0.15	Used marijuana	0.15	PTSD	0.20	Anxiety	0.23
Did not received necessary care last 12 months	0.09	Did not received necessary care last 12 months	0.02	Consumes alcohol ≥1 time weekly	−0.03	Income > $75,000	−0.04
Live births	0.06			Currently employed	−0.04		
PTSD	0.06	Total features	6	Income > $75,000	−0.05	Total features	6
Good self-reported health	−0.01	Percent correct	0.92			Percent correct	0.92
College degree or above	−0.30			Total features	8		
				Percent correct	0.99		
Total features	10						
Accuracy	0.99						

BD, binomial deviance; ME, misclassification error; AUC, area under the ROC (receiver operating characteristic) curve; MSE, mean squared error; MAE, mean absolute error.

Non-clinical characteristics identified as predictors of stroke in females included having a college degree, not receiving necessary healthcare, use of marijuana, good self-reported health, and the number of live births. Non-clinical predictors of male stroke differed from those identified among females and include consuming alcohol, employment, and income.

**Figure 1:**
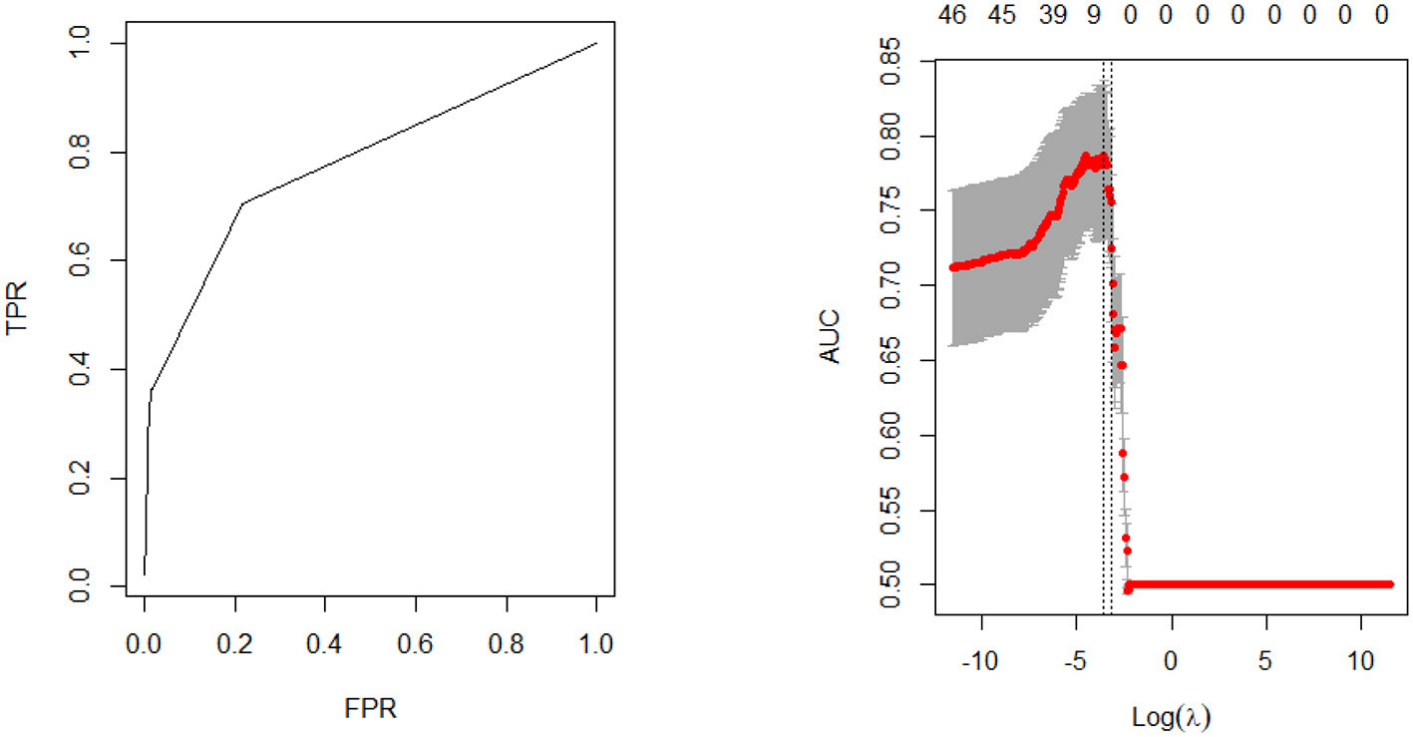
Model performance—male cohort.

**Figure 2:**
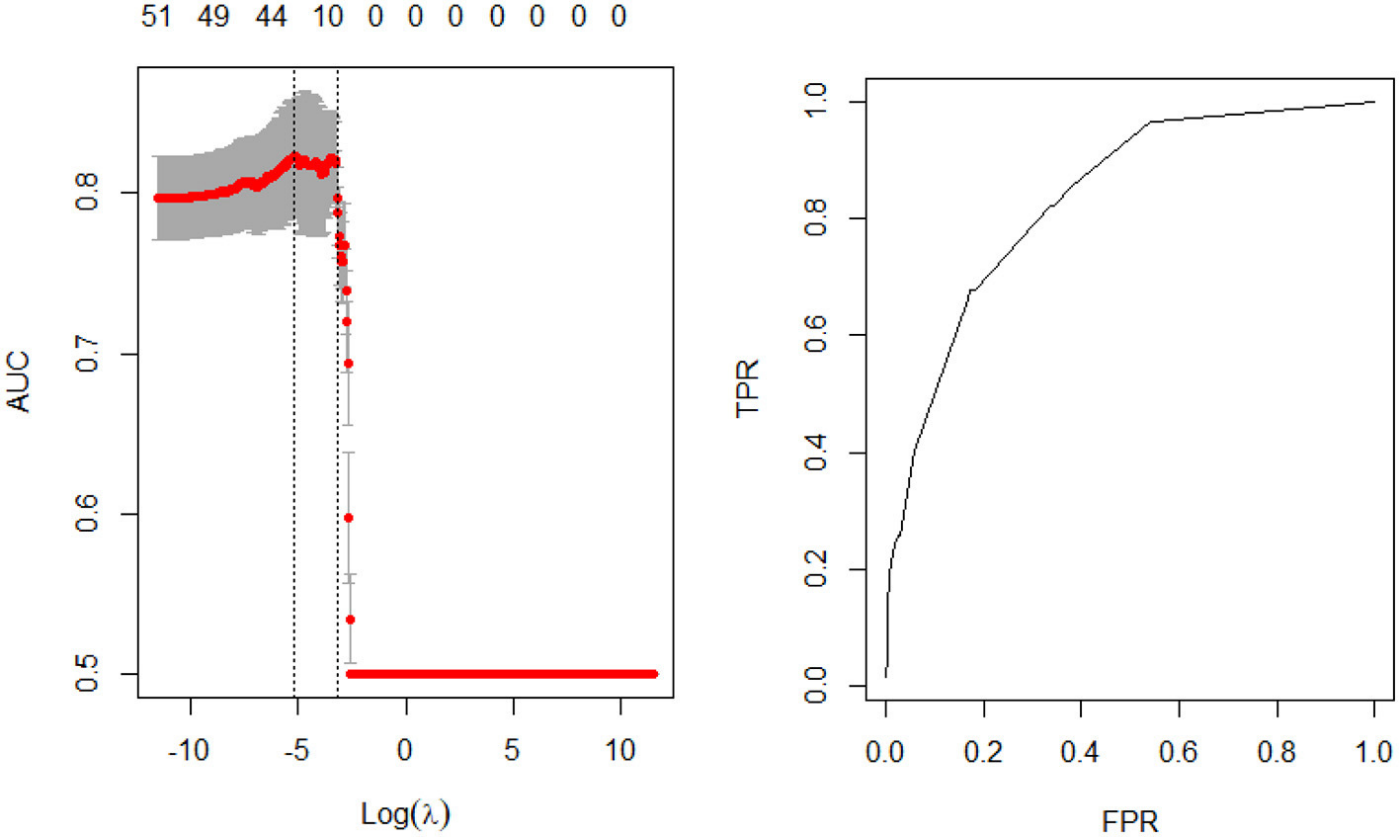
Model performance—female cohort.

Model fit parameters are depicted in Figures 1, 2 for the male and female cohorts respectively. The AUC, deviance, mean error, positive predictive value, and accuracy of each model is also shown in Table 4. The AUC of the unbalanced female model was 0.84 (balanced 0.83) and the unbalanced male model was 0.82 (balanced 0.82). Although there was no difference in AUC between the models, the accuracy was slightly lower for the balanced data models compared to the unbalanced data models (male cohort 0.92 vs. 0.99; female cohort 0.92 vs. 0.99). However, the features identified by both models as well as the order of feature importance was highly similar. Thus, the most important predictors of stroke for each sex remained unchanged.

## Discussion

This study utilized LASSO regression, also known as L1 regularization, to examine clinical and non-clinical predictors of stroke risk among young men and women. LASSO regression is a technique used to estimate the relationships between variables and make predictions by finding a balance between model simplicity and accuracy. It achieves this by adding a penalty term to the regression model, which encourages sparse solutions where some coefficients are forced to be exactly zero. This feature makes LASSO particularly useful for feature selection, as it can automatically identify and discard irrelevant or redundant variables.

Only about one percent of the sample “self-reported” having a stroke which agrees with recent work completed by the US Centers for Disease Control and Prevention showing that approximately one percent of individuals age 18–44 reported having a stroke (Imoisili et al., 2024). Given the age of the population, the incidence of stroke is relatively low. To ensure that the sample imbalance did not bias mode results, data balancing was performed, and the LASSO models were re-estimated on balanced data. The high degree of similarity between these sets of results suggests minimal bias in estimates.

Findings from this analysis showed some similarities as well as some variations in stroke-related risk factors between men and women. For example, kidney disease, diabetes, and post-traumatic stress disorder (PTSD) were predictors of stroke in both men and women. However, specific predictors of stroke among men included heart disease and anxiety whereas women demonstrated a relationship with chronic migraines and depression. Greater variation existed among non-clinical stroke predictors as men were more likely to report alcohol consumption, employment, and income, compared to education, healthcare access, self-reported health, number of live births, and marijuana usage among women.

### Clinical predictors

This study's findings of kidney disease (Krishna et al., 2009) and diabetes (Chen et al., 2016) being predictors of stroke in young adults regardless of sex aligns with previous literature. Additionally, PTSD has been consistently associated with stroke risk (Nanavati et al., 2023). However, these three clinical factors were the only clinical characteristics that were identified as predictors of stroke for both men and women. When considering that sex-related differences in stroke subtypes, etiology, and lateralization have been previously reported (Bonkhoff et al., 2021) and hormonal, physiological, and lifestyle differences contribute to distinct stroke risk factors in men vs. women, we anticipated a greater number and variation and in the clinical predictors for both men and women. For instance Appelros et al. (2009) demonstrated that women were more likely to experience strokes associated with migraines, autoimmune disorders, and hormonal factors, while men more commonly exhibited stroke risks related to lifestyle factors such as smoking and alcohol use. However, this study indicated that there were more predictors of stroke in women compared to men, but lifestyle factors were important determinants among both sexes.

### Non-clinical predictors

The study did not identify any overlap of non-clinical predictors of stroke between men and women. The finding of increased number of live births as a predictor of stroke aligns with previous work demonstrating pregnancy increases risk of stroke (Camargo and Singhal, 2021). Healthcare access and self-reported health emerged as important non-clinical predictors of stroke among women may reflect disparities in the quality of stroke care. Prior studies have shown that women are less likely to receive the same level of evidence-based care for stroke when compared to men with some early stroke care disparities being attributed to differences in initial symptomology among women (Ospel et al., 2023). Additionally, women with stroke were less likely to receive diagnostic services and acute stroke intervention relative to their male counterparts (Roquer et al., 2003). While substance use was observed as predictor in both males and females, the type of substance use differed. Alcohol consumption was a predictor among males whereas marijuana consumption was a risk factor among females. This finding aligns with previous literature demonstrating that alcohol usage and marijuana usage (Jeffers et al., 2024) are associated with stroke risk and men drink more alcohol than women (White, 2020). Lastly, alcohol usage is associated with heart disease (Piano, 2017) suggesting an interrelationship between this non-clinical characteristic and observed variation in clinical characteristics.

Other non-clinical predictors of stroke included employment and income. These findings may be partially explained by traditional reported societal roles related to employment. Although not explored in stroke, sex differences in unemployment and mental health have been observed with men experiencing higher risk of mental health illness with unemployment than women (Artazcoz et al., 2004). Subsequently, the relationship between employment and income and stroke risk in males may occur through neurobiological pathways of stress (Booth et al., 2015). Recently, cultural gender norms have been acknowledged as important to sex and gender differences in health (Bates et al., 2022). Although, we can infer the role of gender inequities such as healthcare access in our study, this study does not include additional variables such as perceptions of masculinity. Clear sex differences in stroke across all ages suggest future work should explore the influence of gender norms on sex differences.

#### Quality of model prediction

In machine learning-based studies of chronic disease such as stroke it is critically important that the techniques and subsequent results be comparable across studies. Therefore, a standard set of metrics are often reported to facilitate these comparisons. In studies applying the LASSO regression, the area under ROC curve (AUC) provides a comprehensive evaluation of the model's prediction performance, ranging from 0.5 to 1 with a value closer to 1 indicating stronger predictive accuracy and better overall model performance. AUC measures overall model performance thereby providing a useful measure for comparing the performance of two different models. In this study we performed 10 × 10-fold cross validation for both the balanced and unbalanced models and found the AUC to be relatively consistent and minimal differences regardless of model (0.82–0.84). We did see that the accuracy of the balanced models was slightly lower than the unbalanced models for both men and women (0.92 vs. 0.99). However, the predictive accuracy overall was high (0.92–0.99)—indicating that these models were equally if not better performing than previous studies exploring stroke diagnosis (Ni et al., 2018). The identification and accuracy of these predictive models underscores the importance of predictive modeling stroke research (Daidone et al., 2024).

### Limitations

Although this study provides valuable information on the predictors of stroke among young men and women, findings must be considered within the context of the following limitations. First, only 149 (59 males and 90 females) of the nearly 12,300 individuals in the ADD Health sample reported having been diagnosed. Small sample sizes have been a consistent concern among stroke studies utilizing machine learning (Lee et al., 2020) and consequently their generalization (Zhi et al., 2024). Second, all information is self-reported and cannot be validated or verified as accurate. Studies have shown that certain health or behavioral conditions can suffer from underreporting, delayed reporting, and incomplete reporting. Further, survey data can also suffer from recency bias, response bias, recall bias, and favorability bias. Third, not all potential predictors of stroke were available in the ADD Health data. For example, the survey did not contain information on coagulation system disorders, antiphospholipid antibody syndrome, or sickle cell anemia. Fourth, the survey employed a complex design and sampling framework that could not be incorporated into the LASSO regression. Fifth, while the LASSO performs both variable selection and regularization to enhance the prediction accuracy and interpretability of the statistical model it produces, it has several limitations including variable selection instability, difficulty handling multicollinearity, and limited variable selection in high dimensional data. Finally, Wave V included a smaller sample than prior waves resulting in smaller samples of all population groups. Additionally, the identified predictors are associations and should not be interpreted as causal factors. Further research is needed to establish causal pathways and underlying mechanisms.

In conclusion, the findings from this study provide valuable insights into the risk factors for stroke among young adults, highlighting the significance of both clinical and behavioral determinants. The application of the LASSO algorithm to a large, nationally representative dataset allowed for the identification of distinct risk profiles for men and women, underscoring the importance of tailored prevention strategies. The modest improvement in model performance with data balancing techniques like SMOTE suggests that addressing data imbalance is beneficial but not transformative. Importantly, this study emphasizes the rising incidence of stroke in younger populations and the need for surveillance and interventions to mitigate this trend. This evidence underscores the necessity of studying men and women separately to inform more effective, personalized prevention and treatment strategies. Future research should focus on refining these models, exploring causal pathways, and developing prevention programs that account for the diversity of identified risk factors.

## Data Availability

The data analyzed in this study is subject to the following licenses/restrictions. This study used ADD Health restricted-use data, which is available only by contractual agreement to certified researchers who commit themselves to maintaining limited access. To be eligible to enter into a contract, researchers must have an IRB-approval letter, security plan for handling and storing sensitive data, and sign a data-use contract agreeing to keep the data confidential. Requests to access these datasets should be directed at: https://data.cpc.unc.edu/projects/2/view.

## References

[B1] Aigner A. Grittner U. Rolfs A. Norrving B. Siegerink B. Busch M. A.. (2017). Contribution of established stroke risk factors to the burden of stroke in young adults. Stroke 48, 1744–1751. 10.1161/STROKEAHA.117.016599 28619986

[B2] Amoah D. Schmidt M. Mather C. Prior S. Herath M. P. Bird M. L.. (2024). An international perspective on young stroke incidence and risk factors: a scoping review. BMC Public Health 24:1627. 10.1186/s12889-024-19134-0 38890645 PMC11186079

[B3] Appelros P. Stegmayr B. Terént A. (2009). Sex differences in stroke epidemiology. Stroke 40, 1082–1090. 10.1161/STROKEAHA.108.540781 19211488

[B4] Arntz R. Rutten-Jacobs L. Maaijwee N. Schoonderwaldt H. Dorresteijn L. van Dijk E.. (2013). Post-stroke epilepsy in young adults: a long-term follow-up study. PLoS ONE 8:e55498. 10.1371/journal.pone.0055498 23390537 PMC3563638

[B5] Artazcoz L. Benach J. Borrell C. Cortès I. (2004). Unemployment and mental health: understanding the interactions among gender, family roles, and social class. Am. J. Public Health 94, 82–88. 10.2105/AJPH.94.1.82 14713703 PMC1449831

[B6] Banks P. Pearson C. (2004). Parallel lives: younger stroke survivors and their partners coping with crisis. Sex Relatsh. Ther. 19, 413–429. 10.1080/14681990412331298009

[B7] Barrio I. Arostegui I. Rodríguez-Álvarez M. X. Quintana J. M. (2017). A new approach to categorising continuous variables in prediction models: proposal and validation. Stat. Methods Med. Res. 26, 2586–2602. 10.1177/0962280215601873 26384514

[B8] Bates N. Chin M. Becker T. (Eds.) (2022). Measuring Sex, Gender Identity, and Sexual Orientation. Washington, DC: National Academies Press. 10.17226/26424 35286054

[B9] Béjot Y. Bailly H. Durier J. Giroud M. (2016). Epidemiology of stroke in Europe and trends for the 21st century. Presse Med. 45, e391–e398. 10.1016/j.lpm.2016.10.003 27816343

[B10] Bennette C. Vickers A. (2012). Against quantiles: categorization of continuous variables in epidemiologic research, and its discontents. BMC Med. Res. Methodol 12:21. 10.1186/1471-2288-12-21 22375553 PMC3353173

[B11] Bonkhoff A. K. Schirmer M. D. Bretzner M. Hong S. Regenhardt R. W. Brudfors M.. (2021). Outcome after acute ischemic stroke is linked to sex-specific lesion patterns. Nat. Commun. 12:3289. 10.1038/s41467-021-23492-3 34078897 PMC8172535

[B12] Booth J. Connelly L. Lawrence M. Chalmers C. Joice S. Becker C.. (2015). Evidence of perceived psychosocial stress as a risk factor for stroke in adults: a meta-analysis. BMC Neurol. 15:233. 10.1186/s12883-015-0456-4 26563170 PMC4643520

[B13] Bukhari S. Yaghi S. Bashir Z. (2023). Stroke in young adults. J. Clin. Med. 12:4999. 10.3390/jcm12154999 37568401 PMC10420127

[B14] Bushnell C. D. Reeves M. J. Zhao X. Pan W. Prvu-Bettger J. Zimmer L.. (2014). Sex differences in quality of life after ischemic stroke. Neurology 82, 922–931. 10.1212/WNL.0000000000000208 24510493 PMC4211921

[B15] Camargo E. C. Singhal A. B. (2021). Stroke in pregnancy. Obstet. Gynecol. Clin. North Am. 48, 75–96. 10.1016/j.ogc.2020.11.004 33573791 PMC7888384

[B16] Chandrabhatla A. S. Kuo E. A. Sokolowski J. D. Kellogg R. T. Park M. Mastorakos P.. (2023). Artificial intelligence and machine learning in the diagnosis and management of stroke: a narrative review of United States Food and Drug Administration-Approved Technologies. J. Clin. Med. 12:3755. 10.3390/jcm12113755 37297949 PMC10253618

[B17] Chen R. Ovbiagele B. Feng W. (2016). Diabetes and stroke: epidemiology, pathophysiology, pharmaceuticals and outcomes. Am. J. Med. Sci. 351, 380–386. 10.1016/j.amjms.2016.01.011 27079344 PMC5298897

[B18] Cramer R. J. Kapusta N. D. (2017). A social-ecological framework of theory, assessment, and prevention of suicide. Front. Psychol. 8:1756. 10.3389/fpsyg.2017.01756 29062296 PMC5640776

[B19] Daidone M. Ferrantelli S. Tuttolomondo A. (2024). Machine learning applications in stroke medicine: advancements, challenges, and future prospectives. Neural. Regen. Res. 19, 769–773. 10.4103/1673-5374.382228 37843210 PMC10664112

[B20] Dev S. Wang H. Nwosu C. S. Jain N. Veeravalli B. John D. A.. (2022). predictive analytics approach for stroke prediction using machine learning and neural networks. Healthc. Anal. 2:100032. 10.1016/j.health.2022.100032

[B21] Dong J. Y. Zhang Y. H. Tong J. Qin L. Q. (2012). Depression and risk of stroke. Stroke 43, 32–37. 10.1161/STROKEAHA.111.630871 22020036

[B22] Ebrahimi R. Lynch K. E. Beckham J. C. Dennis P. A. Viernes B. Tseng C. H.. (2021). Association of posttraumatic stress disorder and incident ischemic heart disease in women veterans. JAMA Cardiol. 6, 642–651. 10.1001/jamacardio.2021.0227 33729463 PMC7970390

[B23] Ekker M. S. Verhoeven J. I. Vaartjes I. van Nieuwenhuizen K. M. Klijn C. J. M. de Leeuw F. E. (2019). Stroke incidence in young adults according to age, subtype, sex, and time trends. Neurology 92, e2444–e2454. 10.1212/WNL.0000000000007533 31019103

[B24] Elwert F. (2013). “Graphical causal models,” in Handbook of Causal Analysis for Social Research, ed. S. L. Morgan (Cham: Springer), 245–273. 10.1007/978-94-007-6094-3_13

[B25] Fan C. Chen M. Wang X. Wang J. Huang B. (2021). A review on data preprocessing techniques toward efficient and reliable knowledge discovery from building operational data. Front. Energy Res. 9:652801. 10.3389/fenrg.2021.652801

[B26] Friedman J. Hastie T. Tibshirani R. (2010). Regularization paths for generalized linear models via coordinate descent. J. Stat. Softw. 33. 10.18637/jss.v033.i01 20808728 PMC2929880

[B27] Harris K. Halpern C. Biemer P. Liao D. Dean S. (2019). Add Health Wave V Documentation: Sampling and Mixed-Mode Survey Design, 2019. Available at: http://www.cpc.unc.edu/projects/addhealth/documentation/guides/ (accessed November 12, 2023).

[B28] Hassan A. Gulzar Ahmad S. Ullah Munir E. Ali Khan I. Ramzan N. (2024). Predictive modelling and identification of key risk factors for stroke using machine learning. Sci. Rep. 14:11498. 10.1038/s41598-024-61665-4 38769427 PMC11106277

[B29] Hernan M. A. (2002). Causal knowledge as a prerequisite for confounding evaluation: an application to birth defects epidemiology. Am. J. Epidemiol. 155, 176–184. 10.1093/aje/155.2.176 11790682

[B30] Imoisili O. E. Chung A. Tong X. Hayes D. K. Loustalot F. (2024). Prevalence of stroke — behavioral risk factor surveillance system, United States, 2011–2022. MMWR Morb. Mortal. Wkly. Rep. 73, 449–455. 10.15585/mmwr.mm7320a1 38781110 PMC11115433

[B31] Irvin J. A. Kondrich A. A. Ko M. Rajpurkar P. Haghgoo B. Landon B. E.. (2020). Incorporating machine learning and social determinants of health indicators into prospective risk adjustment for health plan payments. BMC Public Health 20:608. 10.1186/s12889-020-08735-0 32357871 PMC7195714

[B32] Jeffers A. M. Glantz S. Byers A. L. Keyhani S. (2024). Association of cannabis use with cardiovascular outcomes among US adults. J. Am. Heart Assoc. 13. 10.1161/JAHA.123.030178 38415581 PMC10944074

[B33] Jia J. (2015). Factors related to long-term post-stroke cognitive impairment in young adult ischemic stroke. Med. Sci. Monit. 21, 654–660. 10.12659/MSM.892554 25729006 PMC4354446

[B34] Kino S. Hsu Y. T. Shiba K. Chien Y. S. Mita C. Kawachi I.. (2021). A scoping review on the use of machine learning in research on social determinants of health: trends and research prospects. SSM Popul. Health 15:100836. 10.1016/j.ssmph.2021.100836 34169138 PMC8207228

[B35] Kissela B. M. Khoury J. C. Alwell K. Moomaw C. J. Woo D. Adeoye O.. (2012). Age at stroke. Neurology 79, 1781–1787. 10.1212/WNL.0b013e318270401d 23054237 PMC3475622

[B36] Krawczyk B. (2016). Learning from imbalanced data: open challenges and future directions. Prog. Artif. Intell. 5, 221–232. 10.1007/s13748-016-0094-0

[B37] Krishna P. Naresh S. Krishna G. S. Lakshmi A Vengamma B. Kumar V. (2009). Stroke in chronic kidney disease. Indian J. Nephrol. 19, 5–7. 10.4103/0971-4065.50672 20352003 PMC2845195

[B38] Lasek-Bal A. Kopyta I. Warsz-Wianecka A. Puz P. Łabuz-Roszak B. Zareba K.. (2018). Risk factor profile in patients with stroke at a young age. Neurol. Res. 40, 595–601. 10.1080/01616412.2018.1455367 29577820

[B39] Lee H. Lee E. J. Ham S. Lee H. B. Lee J. S. Kwon S. U.. (2020). Machine learning approach to identify stroke within 4.5 hours. Stroke 51, 860–866. 10.1161/STROKEAHA.119.027611 31987014

[B40] Lee Y. Tsai C. Yen Y. Huang L. K. Chao S. P. Hu L. Y.. (2022). Periodontitis is a potential risk factor for transient ischemic attack and minor ischemic stroke in young adults: a nationwide population-based cohort study. J. Periodontol. 93, 1848–1856. 10.1002/JPER.21-0528 35297043

[B41] Leppert M. H. Burke J. F. Lisabeth L. D. Madsen T. E. Kleindorfer D. O. Sillau S.. (2022). Systematic review of sex differences in ischemic strokes among young adults: are young women disproportionately at risk? Stroke 53, 319–327. 10.1161/STROKEAHA.121.037117 35073188 PMC8852306

[B42] Maaijwee N. A. M. M. Arntz R. M. Rutten-Jacobs L. C. A. Schaapsmeerders P. Schoonderwaldt H. C. van Dijk E. J.. (2015). Post-stroke fatigue and its association with poor functional outcome after stroke in young adults. J. Neurol. Neurosurg. Psychiatry 86, 1120–1126. 10.1136/jnnp-2014-308784 25362090

[B43] Mainali S. Darsie M. E. Smetana K. S. (2021). Machine learning in action: stroke diagnosis and outcome prediction. Front. Neurol. 12:734345. 10.3389/fneur.2021.734345 34938254 PMC8685212

[B44] Maldonado S. López J. Vairetti C. (2019). An alternative SMOTE oversampling strategy for high-dimensional datasets. Appl Soft Comput. 76, 380–389. 10.1016/j.asoc.2018.12.024

[B45] Nanavati H. D. Arevalo A. Memon A. A. Lin C. (2023). Associations between posttraumatic stress and stroke: a systematic review and meta-analysis. J. Trauma Stress 36, 259–271. 10.1002/jts.22925 36987695 PMC10166138

[B46] Ni Y. Alwell K. Moomaw C. J. Woo D. Adeoye O. Flaherty M. L.. (2018). Towards phenotyping stroke: leveraging data from a large-scale epidemiological study to detect stroke diagnosis. PLoS ONE. 13:e0192586. 10.1371/journal.pone.0192586 29444182 PMC5812624

[B47] Ortega Hinojosa A. M. Davies M. M. Jarjour S. Burnett R. T. Mann J. K. Hughes E.. (2014). Developing small-area predictions for smoking and obesity prevalence in the United States for use in Environmental Public Health Tracking. Environ Res. 134, 435–452. 10.1016/j.envres.2014.07.029 25261951

[B48] Ospel J. Singh N. Ganesh A. Goyal M. (2023). Sex and gender differences in stroke and their practical implications in acute care. J. Stroke 25, 16–25. 10.5853/jos.2022.04077 36746379 PMC9911850

[B49] Pearl J. (2009). Causal inference in statistics: an overview. Stat. Surv. 3. 10.1214/09-SS057

[B50] Piano M. R. (2017). Alcohol's effects on the cardiovascular system. Alcohol Res. 38, 219–241. http://www.ncbi.nlm.nih.gov/pubmed/28988575 28988575 10.35946/arcr.v38.2.06 PMC5513687

[B51] Pollock A. St George B. Fenton M. Firkins L. (2014). Top 10 research priorities relating to life after stroke – consensus from stroke survivors, caregivers, and health professionals. Int. J. Stroke 9, 313–320. 10.1111/j.1747-4949.2012.00942.x 23227818

[B52] R Core Team (2021). R: A Language and Environment for Statistical Computing. Vienna: R Foundation for Statistical Computing.

[B53] Reeves M. J. Bushnell C. D. Howard G. Gargano J. W. Duncan P. W. Lynch G.. (2008). Sex differences in stroke: epidemiology, clinical presentation, medical care, and outcomes. Lancet Neurol. 7, 915–926. 10.1016/S1474-4422(08)70193-5 18722812 PMC2665267

[B54] Richmond H. L. Tome J. Rochani H. Fung I. C. H. Shah G. H. Schwind J. S.. (2020). The use of penalized regression analysis to identify county-level demographic and socioeconomic variables predictive of increased COVID-19 cumulative case rates in the State of Georgia. Int. J. Environ. Res. Public Health 17:8036. 10.3390/ijerph17218036 33142755 PMC7663274

[B55] Roquer J. Campello A. R. Gomis M. (2003). Sex differences in first-ever acute stroke. Stroke 34, 1581–1585. 10.1161/01.STR.0000078562.82918.F6 12805490

[B56] Ryder A. L. Cohen B. E. (2021). Evidence for depression and anxiety as risk factors for heart disease and stroke: implications for primary care. Fam. Pract. 38, 365–367. 10.1093/fampra/cmab031 34109973

[B57] Schaapsmeerders P. Maaijwee N. A. M. van Dijk E. J. Rutten-Jacobs L. C. Arntz R. M. Schoonderwaldt H. C.. (2013). Long-term cognitive impairment after first-ever ischemic stroke in young adults. Stroke 44, 1621–1628. 10.1161/STROKEAHA.111.000792 23652272

[B58] Sheth S. A. Giancardo L. Colasurdo M. Srinivasan V. M. Niktabe A. Kan P.. (2023). Machine learning and acute stroke imaging. J. Neurointerv. Surg. 15, 195–199. 10.1136/neurintsurg-2021-018142 35613840 PMC10523646

[B59] Simeonov K. P. Himmelstein D. S. (2015). Lung cancer incidence decreases with elevation: evidence for oxygen as an inhaled carcinogen. PeerJ. 3:e705. 10.7717/peerj.705 25648772 PMC4304851

[B60] Singhal A. B. Biller J. Elkind M. S. Fullerton H. J. Jauch E. C. Kittner S. J.. (2013). Recognition and management of stroke in young adults and adolescents. Neurology 81, 1089–1097. 10.1212/WNL.0b013e3182a4a451 23946297 PMC3795593

[B61] Soun J. E. Chow D. S. Nagamine M. Takhtawala R. S. Filippi C. G. Yu W.. (2021). Artificial intelligence and acute stroke imaging. Am. J. Neuroradiol. 42, 2–11. 10.3174/ajnr.A6883 33243898 PMC7814792

[B62] Sultan S. Elkind M. S. V. (2013). The growing problem of stroke among young adults. Curr. Cardiol. Rep. 15:421. 10.1007/s11886-013-0421-z 24105640 PMC7008634

[B63] Sumner J. A. Cleveland S. Chen T. Gradus J. L. (2023). Psychological and biological mechanisms linking trauma with cardiovascular disease risk. Transl. Psychiatry 13:25. 10.1038/s41398-023-02330-8 36707505 PMC9883529

[B64] Tay J. K. Narasimhan B. Hastie T. (2023). Elastic net regularization paths for all generalized linear models. J. Stat. Softw. 106. 10.18637/jss.v106.i01 37138589 PMC10153598

[B65] Teasell R. W. McRae M. P. Finestone H. M. (2000). Social issues in the rehabilitation of younger stroke patients. Arch. Phys. Med. Rehabil. 81, 205–209. 10.1016/S0003-9993(00)90142-4 10668776

[B66] Tibshirani R. (1996). Regression shrinkage and selection via the Lasso. J. R. Stat. Soc. Ser. B. 58, 267–288. 10.1111/j.2517-6161.1996.tb02080.x

[B67] Torgo L. (2014). An infra-structure for performance estimation and experimental comparison of predictive models in R. arXiv [Preprint]. arXiv:1412.0436. 10.48550/arXiv.1412.0436

[B68] Treger I. Shames J. Giaquinto S. Ring H. (2007). Return to work in stroke patients. Disabil. Rehabil. 29, 1397–1403. 10.1080/09638280701314923 17729085

[B69] Varona J. F. Bermejo F. Guerra J. M. Molina J. A. (2004). Long-term prognosis of ischemic stroke in young adults. J. Neurol. 251, 1507–1514. 10.1007/s00415-004-0583-0 15645352

[B70] Vestling M. Tufvesson B. Iwarsson S. (2003). Indicators for return to work after stroke and the importance of work for subjective well-being and life satisfaction. J. Rehabil. Med. 35, 127–131. 10.1080/16501970310010475 12809195

[B71] White A. (2020). Gender differences in the epidemiology of alcohol use and related harms in the United States. Alcohol Res. Curr. Rev. 40. 10.35946/arcr.v40.2.01 33133878 PMC7590834

[B72] Wiemken T. L. Kelley R. R. (2020). Machine learning in epidemiology and health outcomes research. Annu. Rev. Public Health 41, 21–36. 10.1146/annurev-publhealth-040119-094437 31577910

[B73] Wilson C. M. Biller J. (2004). Ischemic stroke. Adv. Neurol. 92, 1147.

[B74] Yahya T. Jilani M. H. Khan S. U. Mszar R. Hassan S. Z. Blaha M. J.. (2020). Stroke in young adults: current trends, opportunities for prevention and pathways forward. Am. J. Prev. Cardiol. 3:100085. 10.1016/j.ajpc.2020.100085 34327465 PMC8315351

[B75] Zhi S. Hu X. Ding Y. Chen H. Li X. Tao Y.. (2024). An exploration on the machine-learning-based stroke prediction model. Front. Neurol. 15:1372431. 10.3389/fneur.2024.1372431 38742047 PMC11089140

